# An inter-observer assessment of mastoid pneumatization and degree classification using sigmoid sinus: comparing two levels of temporal bone computed tomograms

**DOI:** 10.1007/s00276-023-03130-x

**Published:** 2023-04-06

**Authors:** Okikioluwa Stephen Aladeyelu, Carmen Olivia Rennie, Kurt Schlemmer, Sodiq Kolawole Lawal, Wonder-Boy Eumane Mbatha, Andile Lindokuhle Sibiya

**Affiliations:** 1 Discipline of Clinical Anatomy, School of Laboratory Medicine and Medical Sciences, Nelson R. Mandela School of Medicine Campus, University of KwaZulu-Natal, Durban, South Africa; 2 Discipline of Otorhinolaryngology-Head and Neck Surgery, School of Clinical Medicine, Nelson R. Mandela School of Medicine Campus, University of KwaZulu-Natal, Durban, South Africa; 3 Department of Speech-Language Pathology and Audiology, Faculty of Humanities, University of Pretoria, Pretoria, South Africa; 4 Radiology Department, Inkosi Albert Luthuli Central Hospital Durban, Durban, South Africa; 5 Lake, Smit & Partners Inc. Durban, Durban, South Africa; 6 ENT Department, Inkosi Albert Luthuli Central Hospital Durban, Durban, South Africa

**Keywords:** Pneumatization, Temporal bone, Classification, Sigmoid sinus

## Abstract

**Background:**

The degree of mastoid pneumatization of the temporal bone (TB) has been implicated in the pathogenesis of TB diseases and surgical implications, and planning of a few otologic surgeries. However, there is lack of consensus in the classification of the degree of pneumatization. This study aimed to suggest a simple, quick, and less-burden classification system for assessing and rating the degree of pneumatization by comparing two levels of TB computed tomographs (CTs) using the SS as a reference in an inter-observer assessment among otologists.

**Methods:**

This was a randomized pilot survey among otologists. A questionnaire consisting of different axial CTs of TB taken at two levels: the level of malleoincudal junction (MIJ) and the level of lateral semicircular canal (LSCC), with different pneumatization patterns, was used to assess participants' impressions of the degree of pneumatization. The terms “hypo-,” “moderate,” “good,” and “hyper-” pneumatization were listed as options to rate their impressions on the degree of mastoid pneumatization of the TB images using the SS as a reference structure. Likert scale was used to assess their level of agreement or disagreement with using SS as a reference in evaluating mastoid pneumatization.

**Results:**

Participants who correctly rated images taken at the level of LSCC according to their respective degree of pneumatization were significantly higher (*p* < 0.05) regardless of their year of experience compared to those that correctly rated corresponding images taken at the level of MIJ. A 76% positivity in their level of agreement with the use of sigmoid sinus in evaluating mastoid pneumatization was observed on the Likert-scale chart.

**Conclusion:**

Findings from this study suggest that evaluating air cells around the SS at the level of LSCC on CTs could be easier in assessing and classifying the degree of mastoid pneumatization.

## Introduction

The clinical significance of temporal bone (TB) and its mastoid pneumatization (presence or development of air-filled cavities or air cells) has been highlighted by a number of studies from early 1900 to this present millennium [1–6]. These include playing important roles in surgical intervention in this region of the skull [7, 8], serving as a prognostic factor in chronic and middle ear surgeries [7–9], and providing a protective function to the middle and inner ear in trauma cases [10, 11].

Mastoid pneumatization is clinically relevant in a variety of scenarios. The degree of pneumatization of the mastoid is considered an indirect sign of Eustachian tube function and gas exchange through the mucosa during the first years of life. Early middle ear infections, otitis-prone conditions, and recurrent OME do not allow wide pneumatization of the temporal bone within the first 4 to 6 years of skull growth during childhood. Most acquired cholesteatomas, therefore, present with some degree of sclerosis within the mastoid [12].

Increased pneumatization of the temporal bone is associated with an improved prognosis in otitis media with effusion (OME) [13], as well as higher tympanoplasty graft success rates [14]. It is also relevant to surgical planning for bone conduction implants, active middle ear implants, and even cochlear implantation when assessing the space required for implant placement or predicting access to the middle ear via trans-mastoid, retro-facial approaches [15]. Formerly, a sclerotic mastoid most often required an open (canal wall down) technique, whereas a sufficiently wide and ventilated mastoid led to a combined approach [12]. This is relevant too when considering implantable hearing devices that require an approach through the mastoid, such as the Bone Bridge from Medel, which requires a certain volume of air cells for placement within the mastoid, active middle ear implants that require coupling to ossicles in the epitympanum or cochlear implants that require access through a posterior tympanotomy.

In addition, poorly pneumatized or ‘contracted’ temporal bones may be associated with a low-lying tegmen mastoid, a relatively anteriorly placed sigmoid sinus, or prominent jugular bulb [16, 17], for example, making the surgical approach increasingly precarious [15].

However, the degree and the extent to which the mastoid and entire TB are pneumatized vary among individuals in the same or different population settings [6, 7, 11, 18–21]. According to hereditary theory, the size and the degree of mastoid pneumatization are genetically determined [4]. At the same time, the environmental theory states that how a mastoid is pneumatized solely depends on the degree of pathologic involvement of the middle ear during childhood [20, 22, 23]. Based on these theories, different individuals are expected to exhibit different degrees of mastoid pneumatization in their TBs. Hence, recognizing and classifying different pneumatization patterns are highly important in otology for teaching, diagnosis, and surgical planning.

Very few studies have been identified to classify the degree of pneumatization in TB into Grades, Groups, and Types utilizing computed tomography (CT) scans of the TB [7, 11, 20, 21, 24, 25]. The advent of CT (high resolution) scanning in the 1980s and its uses stirred up diagnostic imaging of the TB and ear structures [26]. This is because CT scanning offers the greatest structural definition of any recently available imaging modality [27, 28]. For instance, TB CT aids in surgical planning and is likely to be useful in evaluating CSF leak risk after surgical base surgery [29].

Of these very few studies, only Han et al. [20] utilized reference structure and specific (anatomical) landmarks to which the degree of mastoid pneumatization was classified. However, the method of Han et al. [20] could be time-consuming and a burden for otologists in a few otologic surgeries, such as primary mastoidectomies, as it involves applying three arbitrary parallel lines angled at 45° in the anterolateral direction, with each line crossing the most anterior point of the sigmoid sinus at the junction with the petrous bone, the most lateral aspect along the transverse plane of the sigmoid groove, and the most posterior point of the sigmoid sinus, respectively.

Furthermore, there was no specific landmark to which the vertical plane for which the three parallel lines are drawn to be angled at 45°, resulting in inaccuracy, especially in axial CT not presented in a straight plane. Therefore, the present study was undertaken to suggest a simple, quick, and less-burden classification system for assessing and rating the degree of pneumatization by comparing two levels of TB computed tomograms using the SS as a reference in an inter-observer assessment among otologists.

## Methods

### Study population

This was a randomized pilot survey among twenty-five (25) cohort otologists comprising registrars and specialists in the Kwazulu-Natal (KZN) province of South Africa. According to the South African Society of Otorhinolaryngology-Head and Neck Surgery (SA Society of ORL-HNS), the sample size used for this pilot survey represents 50% of active and registered Otorhinolaryngologists in KZN, South Africa, excluding emeritus and retirees (Table 1).

**Table 1:** Total number of active and registered otorhinolaryngologists in Kwazulu-Natal, South Africa (Source: Administrative Office, SA Society of ORL-HNS, 2022)

	Kwazulu-Natal	South Africa
Specialists/surgeons	34	226
Registrars	16	70
Total	50	296

### Ethical approval

This was obtained from the Biomedical Research Ethics Committee of the University of KwaZulu-Natal (Protocol Ref. No.: BREC/00002263/2020) and the National Health Research Committee of the Kwazulu-Natal Department of Health (NHRD Ref.: KZ_202102_026).

### The instrument for data collection

A questionnaire that consists of three sections was designed to retrieve data from participants. This questionnaire was constructed after consultation with the Biostatistician of the College of Health Sciences, the University of KwaZulu-Natal, and afterward subjected to face and content validity.

### Questionnaire description

Section "Introduction" consisted of demographic items to retrieve participants’ biodata. Section "Methods" consisted of different axial CT images of TB taken at two levels: the level of malleoincudal junction (MIJ) and the level of the lateral semicircular canal (LSCC), showing different pneumatization patterns. These images were randomly placed on 8 separate pages of the questionnaire, with 4 images per page (4 × 8 = 32 images). The terms “Hypo-, Moderate, Good, and Hyper-pneumatization” were listed as options for participants to rate their impressions on the degree of mastoid pneumatization of the TB images using the SS as a reference structure. The SS was selected as a reference structure according to Shatz and Sadé [30] and Han et al. [20], while the use of qualitative description of the degree of pneumatization was employed in other to strengthen the value of data obtained by standardizing the options into the 4 categories (Hypo, Moderate, Good, and Hyper) for further comparative analysis according to previous studies by Bronoosh et al. [18], Han et al. [20] and as well as recent studies of Aladeyelu et al. [31], Kang et al. [32], and Tan et al. [6]. Section "Results" consisted of a Likert scale to assess the level to which participants agree or disagree with the use of sigmoid sinus as a reference structure in evaluating mastoid pneumatization.

### Image source

All TB CT images used in the questionnaire were sourced retrospectively from the picture archiving and communication systems (PACS) of public hospitals selected for this study in the Kwazulu-Natal province of South Africa. All sourced images were taken using a Multi-Detector row Computed Tomography (MDCT) Scanner (GE Revolution Evo 64 slice, 128 configuration, Milwaukee, Wisconsin, USA).

### Image selections and preparations

Two radiographers were employed in the selection and preparation of images. Images were taken from patients with no ear disease or surgical history. Only scans of patients aged 20 years and above were considered (in other to ensure the completion of pneumatization). Demographically, patients were between the age range 20 and 35 years, of which 18 were males, and 14 were females, belonging to the following South African population groups; Black South African (25; 77%), Indian South African (5; 16%), and White South African (2; 7%).

### Computed tomographs at the MIJ level using SS as a reference

**Fig. 1:**
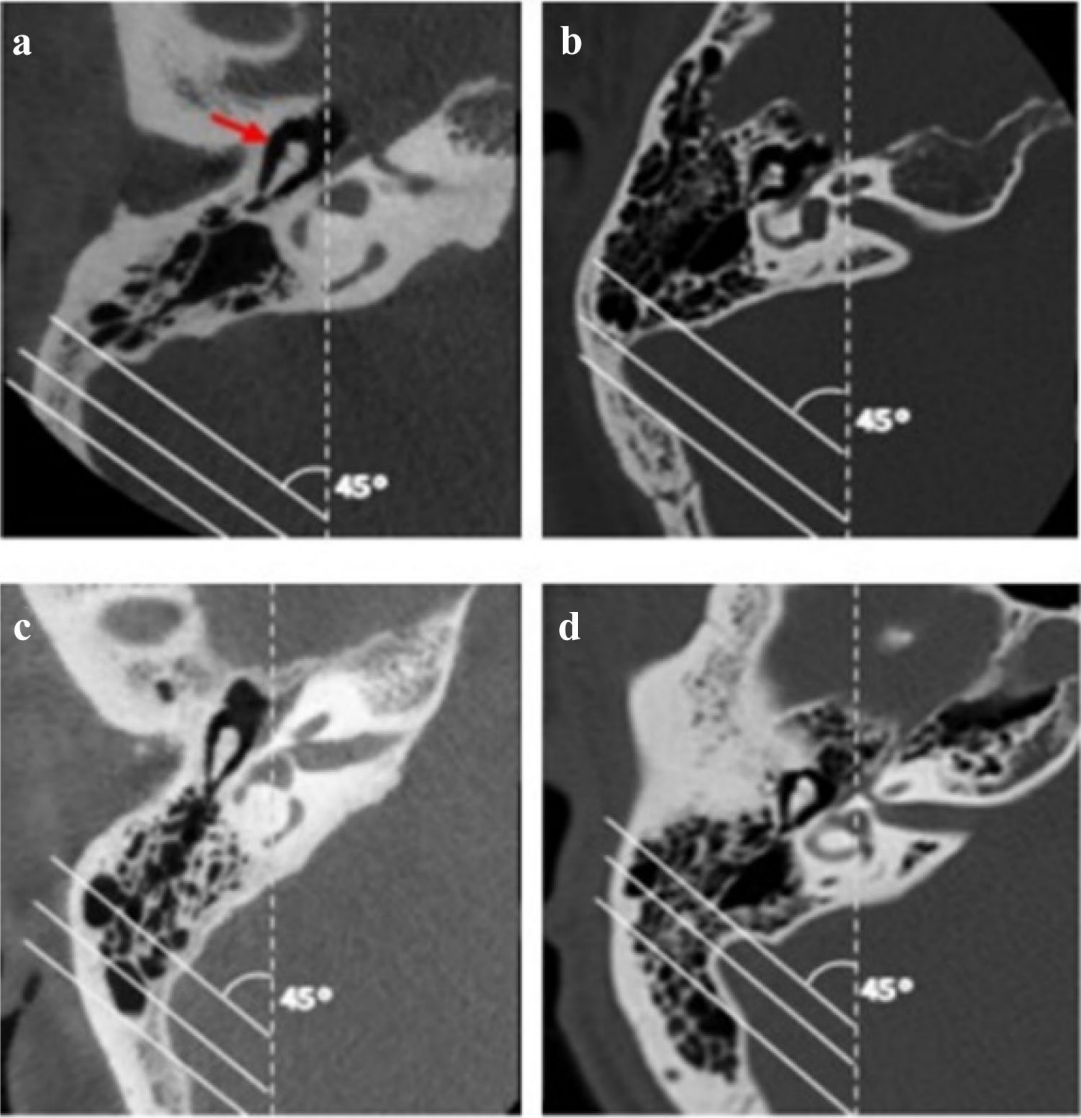
Degrees of pneumatization using SS a reference structure according to Han et al. [10]. On the axial section where the malleoincudal complex (red arrow) appeared as an ice-cream-cone shape. Three parallel lines drawn angled at 45° to the anteroposterior axis (dotted line). **a** hypo-pneumatization, **b** moderate pneumatization, **c** good pneumatization, **d** hyper-pneumatization (Color figure online)

**Fig. 2:**
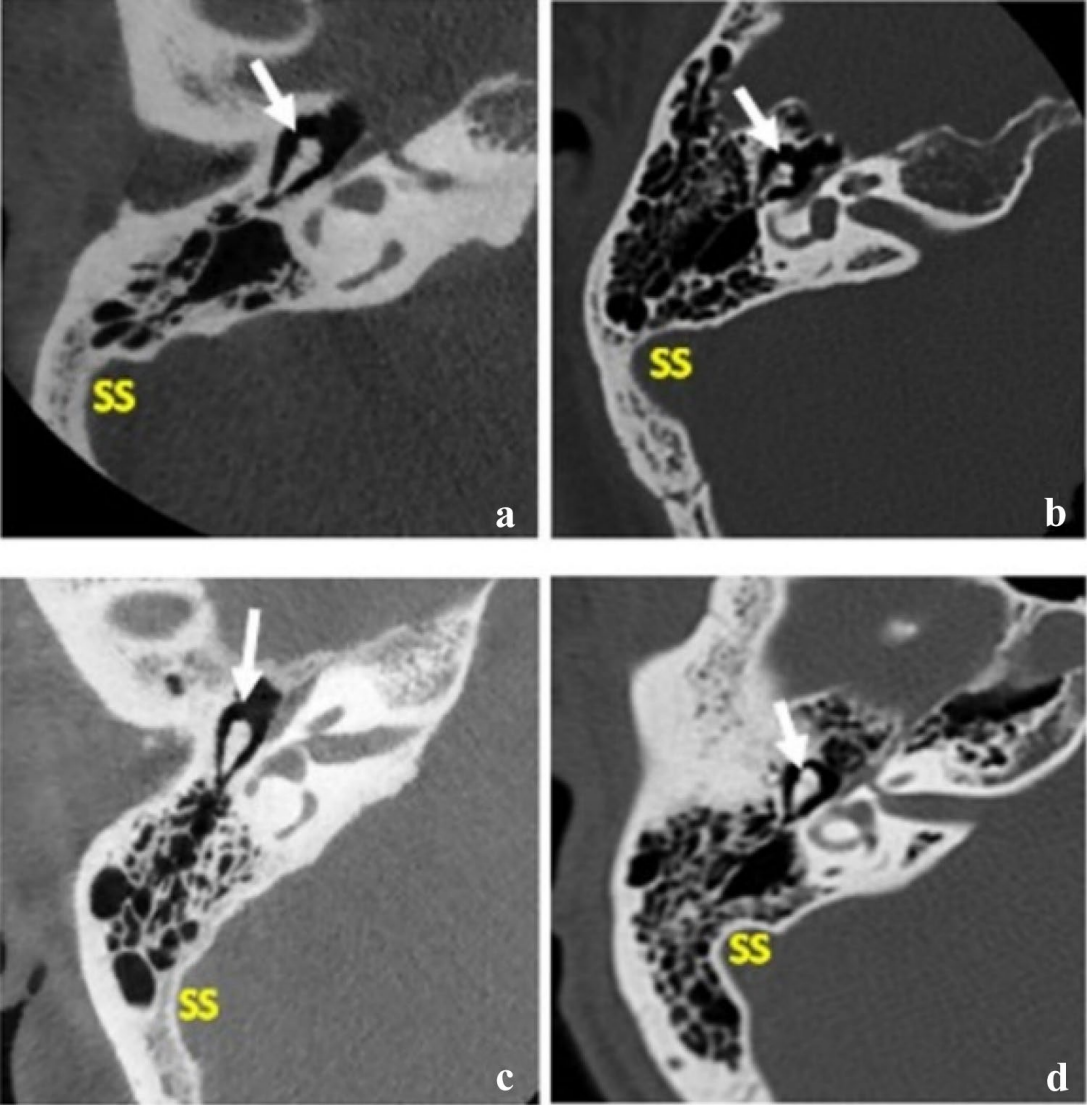
A representation of axial CT images taken at the level of the MIJ (white arrow) used in the questionnaire showing different degrees of pneumatization after removing the arbitrary lines. **a** hypo-pneumatization; **b** moderate pneumatization; **c** good pneumatization; **d** hyper-pneumatization. *SS* sigmoid sinus (Color figure online)

Here, images were prepared using the classification system proposed by Han et al. [10]. At the axial section in which the malleoincudal complex appears like an ice-cream cone shape, three parallel lines were applied in the anterolateral direction angled at 45°, with each crossing the most anterior point of the sigmoid sinus at the junction with the petrous bone, the most lateral aspect along the transverse plane of the sigmoid groove, and the most common posterior point of the sigmoid sinus, respectively (Fig. 1). With the application of these lines, the degree of mastoid pneumatization was classified into four groups: Hypo-pneumatization—pneumatization that extends to the line drawn at the most anterior aspect of the SS (Fig. 1a); Moderate pneumatization—pneumatization that extends to the space between arbitrary lines drawn at the most anterior point and most lateral aspect of the sigmoid sinus (Fig. 1b); Good pneumatization—pneumatization that extends to the space between the lines drawn at the most lateral region and the most posterior point of the SS (Fig. 1c) and; Hyper-pneumatization—pneumatization that extends postero-laterally beyond the line drawn at the posterior point of the sigmoid sinus (Fig. 1d). After that, the arbitrary lines were removed before images were placed in the questionnaire (Fig. 2).

### Computed tomographs at the LSCC level using SS as a reference

**Fig. 3:**
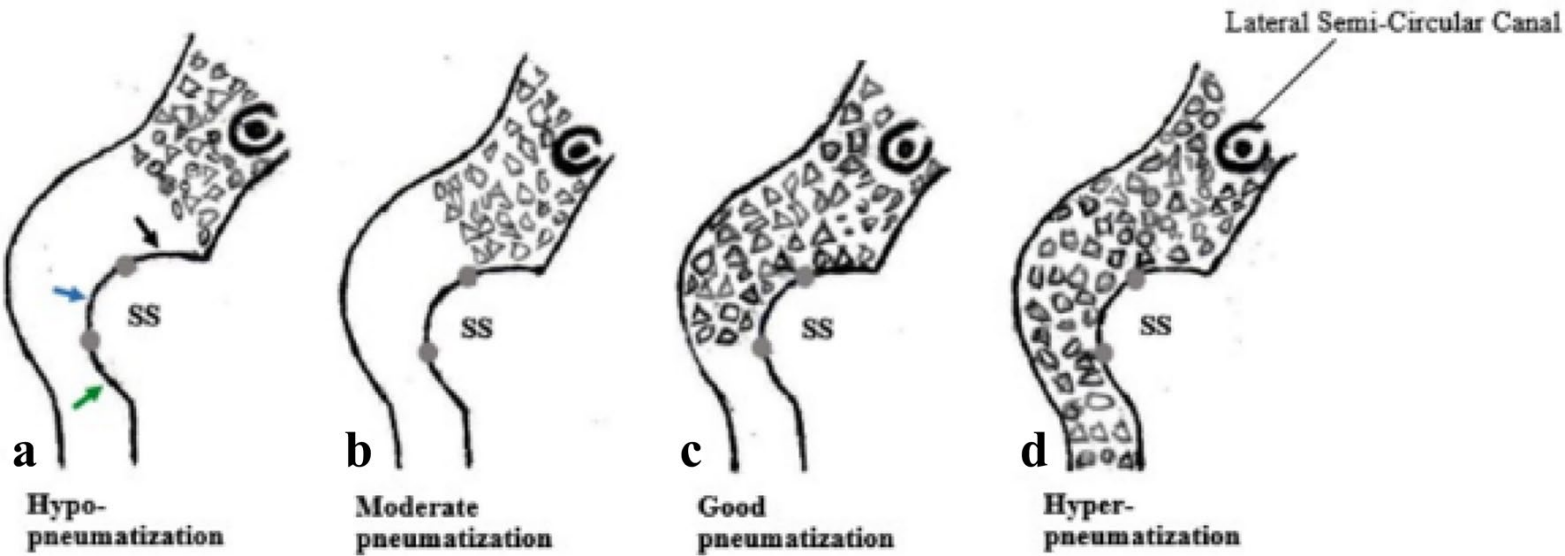
Diagram showing the proposed classification system. SS—sigmoid sinus. Gray dots—2 imaginary dots. Black arrow—superior–lateral 1/3 segment. Blue arrow—middle–lateral 1/3 segment. Green arrow—inferolateral side 1/3 segment (Color figure online)

**Fig. 4:**
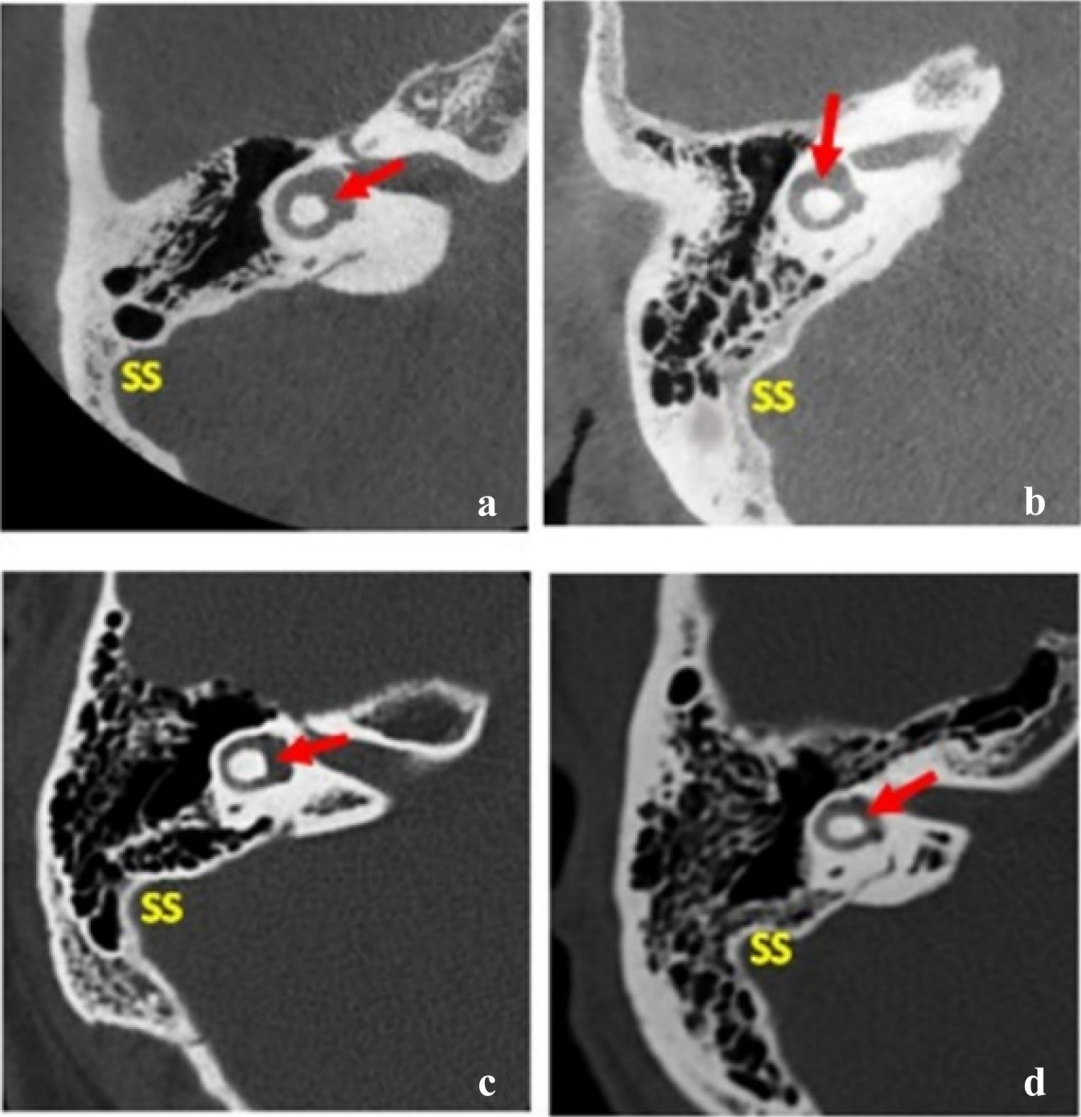
A representation of axial CT images taken at the level of the LSCC (red arrow) used in the questionnaire showing different degrees of pneumatization after dividing the lateral convex or semicircular-shaped line/border of the sigmoid sinus into three equal segments using two imaginary dots. **a** hypo-pneumatization; **b** moderate pneumatization; **c** good pneumatization; d) hyper-pneumatization. *SS* sigmoid sinus (Color figure online)

Here, images were prepared using this study's present suggested classification system*.* On the axial CT image at the level of the lateral semicircular canal (LSCC) level, the lateral convex or semicircular-shaped line/border of the sigmoid sinus was divided into three equal segments by two imaginary dots (gray dots); superior-lateral 1/3 (black arrow), lateral 1/3 (blue arrow), and inferolateral 1/3 (green arrow) (Fig. 3). The degree of mastoid pneumatization was classified as follows: hypo-pneumatization—when no air cells are found around any of the three segments of the lateral convex border of the sigmoid sinus (Fig. 3a); Moderate pneumatization—when air cells are found only in relation to the superior–lateral 1/3 segment and limited to this segment (Fig. 3b); Good pneumatization—when air cells are found in relation to both the superior–lateral 1/3 and the lateral 1/3 segments (Fig. 3c); Hyper-pneumatization—when air cells are found in relation to all three segments and even extend further after the inferolateral 1/3 (Fig. 3d). In addition, this classification system was verified by comparing each degree of mastoid pneumatization of the present suggested classification system with their corresponding degree of mastoid pneumatization in the classification system proposed by Han et al. [10] before using it to select CT images that were put in the questionnaire (Fig. 4).

### Statistical analysis

The statistical analysis was conducted using R Statistical computing software of the R Core Team, 2020, version 3.6.3. Participants who correctly rated the degree of pneumatization of images taken at any of the two levels were expressed in counts and percentages. The mean and the mode were calculated for the Likert scale. Because of the small sample size, Fisher’s exact test was used to compare the percentage of participants who correctly rated images taken at the level of the LSCC to the percentage of participants who correctly rated images taken at the level of the MIJ. Statistical significance was set at *p* < 0.05. Furthermore, a Likert Chart was plotted in a Python programming environment using Google Colab.

## Results

A total of twenty-five questionnaires were distributed, and a 100% return rate was achieved. Among the otologists who participated in the study, 64% were specialists, while 32% were registrars. In their year of experience as otologists, 36% have had 1–5 years of experience, 28% have had 6–10 years of experience, and 36% have had more than 11 years of experience. In their relative otology burden or TB-related surgeries monthly, 20% of the respondents encounter < 20% in a month, 56% encounter between 21 and 50% in a month, and 24% encounter > 51% in a month.

The result presented in Table 2 showed the percentage of participants that correctly rated the degree of pneumatization of axial images taken at the level of MIJ and LSCC. The total number of participants who correctly rated all images taken at the level of LSCC according to their respective degree of pneumatization was above 50%. For moderate, good, and hyper-pneumatized images, participants who correctly rated at the level of LSCC were more than participants who correctly rated these images at the level of MIJ, with significant differences of *p* < 0.05. Participants who correctly rated hypo-pneumatized images taken at the level of MIJ were higher. Still, there was no significant difference (*p* = 0.056) when compared to the number of participants who correctly rated hypo-pneumatized images taken at the level of LSCC.

**Table 2:** Comparison between overall participants who correctly rated images at LSCC level and MIJ level

	Axial CTs sections taken at the level of LSCC No. of participants (%)	Axial CTs sections taken at the level of MIJ No. of participants (%)	*p* value
Hypo-pneumatized images	14 (56)	19 (76)	0.056
Moderately pneumatized images	13 (52)	3 (12)	0.014*
Good pneumatized images	18 (72)	5 (20)	0.003*
Hyper-pneumatized images	16 (64)	6 (24)	0.012*

*p < 0.05 shows significant differences in the total number of participants who correctly rated images taken at the level of LSCC when compared to images taken at the level of MIJ at different degrees of pneumatization

As per professional status, all participants in the two professional statuses contributed to the total percentage of participants who correctly rated all axial CT images taken at the level of LSCC, but specialists contributed more. The same was observed in the years of experience of participants, with the participants with the highest work experienced participants (> 11 years) contributing more (Table 3). As for axial CT images taken at the MIJ level, not all participants, as per professional status and years of experience, contributed to the overall percentage of participants who correctly rated images taken at this level (Table 4)**.** Furthermore, comparing participants with the same year of experience in the rating of images at the LSCC level and MIJ level showed that in the different groups of year experience, more participants were able to rate correctly images taken at the level of LSCC (Table 5).

**Table 3:** Percentage of participants who correctly rated images of axial sections taken at LSCC level based on professional demographics

Images	Percentage of participants (%)	Percentage as per professional status		Percentage as per years of experience		
		Specialist (%)	Registrar (%)	1–5 (%)	6–10 (%)	> 11 (%)
Hypo-pneumatized	56.0	36	20	12	20	24
Moderately pneumatized	52.0	40	12	12	12	28
Good pneumatized	72.0	52	14	20	20	32
Hyper-pneumatized	64.0	52	18	12	20	32

**Table 4:** Percentage of participants who correctly rated images of axial sections taken at MIJ level based on professional demographics

Images	Percentage of participants (%)	Percentage as per professional status		Percentage as per years of experience		
		Specialist (%)	Registrar (%)	1–5 (%)	6–10 (%)	> 11 (%)
Hypo-pneumatized	76.0	48	28	28	20	28
Moderately pneumatized	12.0	12	0	0	0	12
Good pneumatized	20.0	20	0	4	12	4
Hyper-pneumatized	24.0	20	0	4	4	16

**Table 5:** Comparison between participants who correctly rated images at LSCC level and MIJ level in relation to their year of experience

Images	Percentage as per year of experience between LSCC level and MIJ level					
	1–5 years		6–10 years		> 11 years	
	LSCC (%)	MIJ (%)	LSCC (%)	MIJ (%)	LSCC (%)	MIJ (%)
Hypo-pneumatized	12	28	20	20	24	28
Moderately pneumatized	12	0	12	0	28	12
Good pneumatized	20	4	20	12	32	4
Hyper-pneumatized	12	4	20	4	32	16

**Fig. 5:**
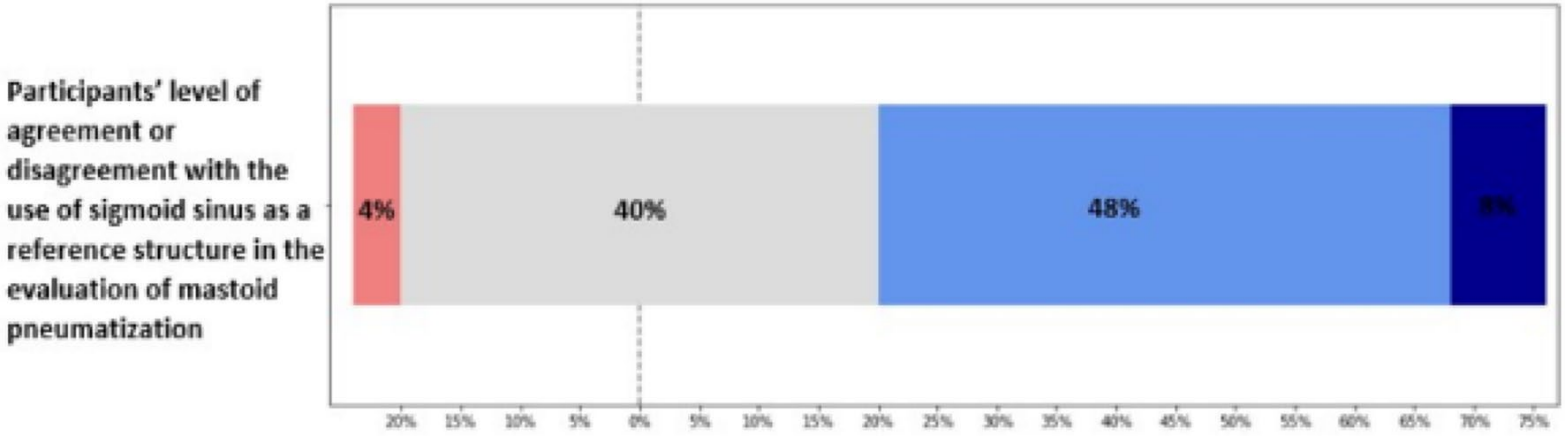
Showing the level to which participants agree or disagree with the use of sigmoid sinus as a reference structure in evaluating mastoid pneumatization

The level to which participants agree or disagree with using SS as a reference structure in the evaluation of mastoid pneumatization is presented in Fig. 5. The Likert scale was rated from 1 to 5: 1- strongly disagree, 2- disagree, 3- neutral, 4- agree, and 5- strongly agree. Statistically, participants' mean and mode responses were 3.6 and 4, respectively. The Likert-scale 4 (agree) was observed to have the highest frequency, with 48%. Generally, the Likert chart showed 76% positivity and 24% negativity.

## Discussion

The degree of mastoid pneumatization of the TB is of various clinical significance, including implications on the pathogenesis of TB diseases and surgical implications and planning of a few otologic surgeries, such as primary mastoidectomies [8, 17, 29, 32]. It also can give the life history of an individual TB exposure to diseases and Eustachian tube function. This is because, on the one hand, a small mastoid pneumatization is a prerequisite of middle-ear disease. On the other hand, the extent of pneumatization mostly depends on the degree of pathological involvement of the middle ear during childhood [20, 23]. Despite these, there is still a lack of consensus about the classification of TB pneumatization among otologists.

The present study utilized axial CT images of temporal bone taken at the level of MIJ and LSCC to conduct an inter-observer assessment of the pneumatization of the temporal bone images among otologists utilizing the classification terms provided to rate the degree of mastoid pneumatization of the temporal bone using SS as a reference. The use of SS as a reference conforms to the study of Han et al. [20] and Shatz and Sadé [30].

More otologists were able to correctly rate axial CT images of TB taken at the level of LSCC according to the respective degree of mastoid pneumatization (with participants with the highest work experience and professional status contributing more), which was significantly higher when compared to the number of otologists who correctly rated corresponding axial CT images of TB taken at the level of the MIJ. It is worth noting that participants were blinded to the fact that axial CT images included in the survey were limited to 2 levels. In addition, the study also found that otologists with the lowest year of experience and professional status could correctly rate the degree of mastoid pneumatization of all axial CT images taken at the level of LSCC. This, however, reflects the simplicity and preciseness of this study's suggested new classification system, as less experienced doctors could also utilize this system in classifying the degree of mastoid pneumatization of the TB. It also reflects a high correlation between mastoid pneumatization and sigmoid sinus at this level, as reported by Shatz and Sadé [30]. This, however, could be easily utilized when reviewing CT images of TB prior to surgery as well-pneumatized TBs are possibly skeletonizing or exposing critical structures, such as facial nerve and labyrinth.

The Likert chart observed a high positivity in participants’ level of agreement with the use of SS in evaluating mastoid pneumatization. This, however, agrees with Han et al. [20] on utilizing the SS as a reference structure since the evaluation of air cells around the sigmoid sinus could interpret the entire pneumatization of the TB. In addition, the extent of mastoid air cells was also reported by Yamakami et al. [29] and Hindi et al. [33] to correlate significantly to the pneumatization of the petrous apex and other parts of the TB.

Hence, estimating the degree of mastoid pneumatization using SS at the level of the LSSC as a reference point during surgical planning can be easily utilized by otologists in analyzing critical areas or structures that might be affected by varying degrees of pneumatization, such as the proximity of the facial nerve as well as the articular eminence of the temporo-mandibular joint (TMJ) to the canal in procedures, such as canaloplasty or subtotal petrosectomy [34, 35], as well as approaches to the sinus tympani and its relationship to the facial nerve and posterior semicircular canal (PSCC) [36].

## Conclusion

This study conducted an interobserver assessment of mastoid pneumatization and classification of its degree of pneumatization using sigmoid sinus by comparing axial CT images taken at the level of MIJ and the level of LSCC among otologists. The result from this survey revealed that evaluating air cells around the SS on TB axial computed tomograms taken at the level of the LSCC was more precise among the participants regardless of their professional status and year of experience. This study hopes that the suggested new classification system (Fig. 3) could be generalized among otologists as it could be easier and less burdensome, assisting otologists in reviewing mastoid pneumatization before surgery in other to analyze critical structures that might be at risk.

### Suggestions for future study

It is noteworthy that, following a thorough literature search, this is a novel approach reproducible in developing a classification system for the degree of mastoid pneumatization. Hence, the authors would like to allude to future studies such as: (i) Testing the repeatability of this suggested classification system in a new group of clinicians (including radiologists and surgeons) who are taught the classification beforehand and correlate with clinical disease entities such as cholesteatoma or otitis media effusion or chronic otitis media. (ii) Conducting a survey among clinicians to characterize the degree of pneumatization at different levels of CT images, including the MIJ and LSCC levels.

## Data Availability

Available on request.
